# Incidence rates of tendinopathies and non-traumatic tendon ruptures in Hong Kong: a 25-year epidemiological study using big data from public hospitals

**DOI:** 10.1038/s41598-025-33465-x

**Published:** 2026-01-12

**Authors:** Kenney Ki Lee Lau, Jonathan Patrick Ng, Samuel Ka Kin Ling, Michael Tim Yun Ong, Patrick Shu Hang Yung, Pauline Po Yee Lui

**Affiliations:** 1 Department of Orthopaedics and Traumatology, The Chinese University of Hong Kong, Hong Kong, China; 2 InnoHK Center for Neuromusculoskeletal Restorative Medicine, Hong Kong Science Park, Hong Kong, China; 3 Department of Orthopaedics and Traumatology, Prince of Wales Hospital, Hong Kong, China

**Keywords:** Tendinopathy, Non-traumatic tendon rupture, Epidemiology, Incidence, Population-based, Hong Kong, Anatomy, Diseases, Health care, Medical research

## Abstract

Tendinopathy affects a substantial proportion of individuals and imposes a notable burden on healthcare systems. Its management poses considerable challenges, largely due to the limited availability of effective treatments, some of which may eventually lead to tendon tears. Despite its clinical importance, epidemiological research on tendinopathic conditions remains sparse and somewhat contentious. Consequently, there is an urgent need for comprehensive data to inform prevention strategies and optimize resource allocation. This study investigated the incidence of tendinopathy and non-traumatic tendon rupture in the Hong Kong population, using medical records from public hospitals. Data were sourced from the Clinical Data Analysis and Reporting System (CDARS). Patients with tendinopathy or non-traumatic tendon rupture were identified using the International Classification of Diseases, Ninth Revision, Clinical Modification (ICD-9-CM) from 2000 to 2024. The overall, age-standardized, and sex-standardized annual incidence rates, as well as the age-specific and sex-specific annual incidence rates, were presented. Our study involved 36,970 patients across 47 public hospitals. The total annual incidence rate for the 17 tendinopathies and non-traumatic tendon ruptures included was 21.05 ± 7.08 cases per 100,000 persons. Incidence rate for the 10 tendinopathies was 15.48 ± 6.33, whereas for the 7 non-traumatic tendon ruptures was 5.57 ± 0.93. Tendinopathy had the highest overall incidence among cases of lateral epicondylitis, at 8.84 ± 3.31 cases per 100,000 persons, followed by Achilles tendinopathy at 3.09 ± 1.59. Regarding non-traumatic tendon rupture, rotator cuff tendon rupture had the highest average incidence at 1.90 ± 0.87 cases per 100,000 persons, followed by Achilles tendon rupture at 1.73 ± 0.37. Older individuals (aged 40–59) showed higher rates of most tendinopathies, except for patellar and Achilles tendinopathies predominantly affected in youngsters. Females had higher rates of several tendinopathies, while males had higher rates of patellar tendinopathy and various tendon ruptures. Our findings highlight several key tendon conditions for targeted prevention, with a focus on middle-aged adults and especially females for tendinopathies and males for non-traumatic tendon ruptures. Primary care providers are encouraged to facilitate earlier referrals for lateral epicondylitis and Achilles tendinopathy, while orthopaedic surgeons are advised to consider earlier interventions for these conditions. Additionally, emergency staff may consider additional training in the management of commonly encountered tendon conditions, such as rotator cuff tears and Achilles tendon ruptures.

## Introduction

Tendinopathy refers to a multifaceted disorder characterized by tendon pain^1^. While overuse and repetitive strain are major contributors to its development^2^, other factors like age^3^, specific prescriptions^4^, medical conditions^5^, and genetic variations^6^, can also play a role. However, managing tendinopathy can be challenging as many therapies yield limited treatment success^7^, and some patients may ultimately suffer from tendon tears due to aggravated degeneration^8,9^. Instead, tendon rupture denotes a visible discontinuity in the tendon^1^. Significant trauma can cause complete ruptures in healthy tendons, while minor traumatic events can also cause tendon ruptures, but these usually happen in tendinopathy^10,11^. Despite several key intervention studies that have explored cures for tendinopathy and tendon rupture^12–14^, research evaluating their epidemiology remains scarce and controversial^15–17^.

Specifically, a crucial drawback of the existing literature is that most studies investigating incidence measures have focused on lower limb tendinopathy^18–23^and tendon ruptures^24–33^. Consequently, healthcare resource allocation may have been misguided as the epidemiological factors affecting upper limb tendons remain largely uncharted^34^. It is plausible that issues related to the upper extremity could be more serious^10^. A better comprehension of the full scope of tendinopathies and non-traumatic tendon ruptures in commonly affected body regions can help clinicians, scientists, and policymakers effectively target the areas that require proper attention. Moreover, some previous incidence studies have primarily examined specific populations, such as athletes^20,23^and soldiers^25,29^, leaving a gap in understanding its impact on the general public. While certain groups of physically active individuals may be at elevated risk of developing tendon pathologies^2,7,10^, the general population is also affected by these tendon conditions. Furthermore, there is a paucity of studies scrutinizing the incidence of non-traumatic tendon ruptures. Given the above, there is a pressing need to study the incidences of tendinopathy and non-traumatic tendon rupture across body regions with a representative sampling.

Therefore, our objective was to assess the overall, age-standardized, and sex-standardized annual incidence of tendinopathy and non-traumatic tendon rupture in the shoulder, elbow, hand and wrist, hip, knee, as well as the foot and ankle, from 2000 to 2024. This analysis was based on electronic medical records from all local public hospitals, which represent 75% of all hospitals in Hong Kong. Additionally, we sought to evaluate the age-specific and sex-specific annual incidence of various tendinopathies and non-traumatic tendon ruptures. Understanding these incidence figures enables the identification of anatomical regions and specific populations at heightened risk, thereby promoting the development of targeted preventive strategies. More importantly, resource allocation in public hospitals could be optimized by prioritizing the management of the most severe types of tendinopathy and non-traumatic tendon rupture, thus alleviating the burden on the public healthcare system.

## Methods

## Study design

This was a retrospective cohort study with 25 years of patient data. The study protocol was approved by the Joint Chinese University of Hong Kong and New Territories East Cluster Clinical Research Ethics Committee (reference number: 2025.363). It has been performed in accordance with the Declaration of Helsinki. Informed consent was waived because the study did not involve direct patient contact and utilized anonymized data.

## Data source

Patient data were retrieved from the Clinical Data Analysis and Reporting System (CDARS), a territory-wide public hospital database developed by the Hospital Authority (HA) in Hong Kong^35,36^. As the statutory body overseeing all government hospitals, HA provides publicly funded healthcare services at primary, secondary, and tertiary levels to all residents in Hong Kong. CDARS encompasses extensive patient-specific data, including demographic information, payment methods, diagnoses, prescription and dispensing details, admission and discharge records, and laboratory test results. It includes comprehensive records of inpatient, outpatient, and emergency department admissions across its clinics and hospitals, dating back to 1993. To ensure patient privacy, all data are anonymized by de-identifying patient identities and generating unique patient reference numbers, facilitating secure and efficient data retrieval.

## Case identification

Target patients were identified using the International Classification of Diseases, Ninth Revision, Clinical Modification (ICD-9-CM) diagnostic codes (Table 1). For the diagnosis of tendinopathy, we included the following codes: 726.10 (disorders of bursa and tendon in shoulder region), 726.11 (calcifying tendinitis of shoulder), 726.12 (bicipital tenosynovitis), 726.31 (medial epicondylitis), 726.32 (lateral epicondylitis), 726.8 (other peripheral enthesopathy), 726.5 (enthesopathy of hip region), 726.64 (patellar tendinitis), 726.61 (pes anserinus tendonitis or bursitis), 726.71 (Achilles bursitis or tendinitis), 726.72 (tibialis tendinitis), and 726.79 (other enthesopathy of ankle and tarsus). For the diagnosis of non-traumatic tendon rupture, we involved the following codes: 727.61 (complete rupture of rotator cuff), 727.62 (non-traumatic rupture of tendons of biceps long head), 727.63 (non-traumatic rupture of extensor tendons of hand and wrist), 727.64 (non-traumatic rupture of flexor tendons of hand and wrist), 727.66 (non-traumatic rupture of patellar tendon), 727.65 (non-traumatic rupture of quadriceps tendon), 727.67 (non-traumatic rupture of Achilles tendon), and 727.68 (non-traumatic rupture of other tendons of foot and ankle). All patients diagnosed with tendinopathy or non-traumatic tendon rupture from the 47 local public hospitals were included in the study. In case of multiple diagnostic records within a category, only the first episode was counted. When a record of tendinopathy was observed after a non-traumatic tendon rupture, it was not classified as a tendinopathy case. Only tendinopathy records documented prior to a non-traumatic tendon rupture were considered. The data retrieval period spanned from January 1, 2000, to December 31, 2024.

**Table 1:** Diagnostic codes included for database retrieval.

Tendinopathy	Non-traumatic tendon rupture
Shoulder	
726.10 Disorders of bursa and tendon in shoulder region • Supraspinatus tendinitis • Tendinitis of shoulder	727.61 Complete rupture of rotator cuff • Complete rupture of rotator cuff, nontraumatic • Degenerative rupture of rotator cuff • Nontraumatic supraspinatus tear • Nontraumatic complete rupture of rotator cuff
726.11 Calcifying tendinitis of shoulder • Calcifying tendinitis of shoulder	
726.12 Bicipital tenosynovitis • Biceps tendinitis	727.62 Nontraumatic rupture of tendons of biceps (long head) • Rupture of long head of biceps, nontraumatic • Nontraumatic rupture of long head biceps
Elbow	
726.31 Medial epicondylitis • Medial epicondylitis	
726.32 Lateral epicondylitis • Lateral epicondylitis • Golfer’s elbow • Tennis elbow	
Hand and Wrist	
726.8 Other peripheral enthesopathy • Tendonitis of finger	727.63 Nontraumatic rupture of extensor tendons of hand and wrist • Nontraumatic rupture of extensor tendons of hand and wrist • Degenerative rupture of extensor tendon of hand • Nontraumatic rupture extensor tendon of hand and wrist
	727.64 Nontraumatic rupture of flexor tendons of hand and wrist • Nontraumatic rupture of flexor tendons of hand and wrist • Nontraumatic rupture flexor tendon of hand and wrist
Hip	
726.5 Enthesopathy of hip region • Gluteal tendinitis • Psoas tendinitis	
Knee	
726.64 Patellar tendinitis • Patellar tendonitis • Patellar tendinitis	727.66 Nontraumatic rupture of patellar tendon • Nontraumatic rupture of patellar tendon
726.61 Pes anserinus tendonitis or bursitis • Pes anserinus tendonitis	
	727.65 Nontraumatic rupture of quadriceps tendon • Nontraumatic rupture of quadriceps tendon • Nontraumatic rupture of quadriceps tendon in thigh
Foot and Ankle	
726.71 Achilles bursitis or tendinitis • Achilles tendonitis • Achilles tendinitis	727.67 Nontraumatic rupture of Achilles tendon • Nontraumatic rupture of Achilles tendon
726.72 Tibialis tendinitis • Tibialis tendonitis • Tibialis posterior tendonitis	727.68 Nontraumatic rupture of other tendons of foot and ankle • Nontraumatic rupture of the tendon of foot and ankle
726.79 Other enthesopathy of ankle and tarsus • Peroneal tendinitis	

## Outcome measures

Primary outcome of the present study was the annual incidence rate, calculated using population estimates from the Census and Statistics Department of the Hong Kong government^37^. For each year, the number of cases of tendinopathy or non-traumatic tendon rupture was divided by the total local population for that year. This calculation produced a crude incidence rate expressed as the number of cases per 100,000 individuals. Further, the age-specific and sex-specific rates are calculated for each respective age and sex group by dividing the number of cases in a particular subgroup by the corresponding population. Regarding the age-standardized and sex-standardized rates, each age-specific or sex-specific rate is multiplied by the proportion of the corresponding population in the year corresponding to the pertinent age or sex group (standard population weight), and the standardized rate is obtained by summing the resulting values.

## Statistical analysis

Data analysis was performed using the Statistical Product and Service Solutions (SPSS) software version 30.0, with a significance level of 0.05 (two-tailed). Incidence rates of tendinopathies and non-traumatic tendon ruptures were reported descriptively. Overall, age-standardized, and sex-standardized measures are reported as annual incidence rates, calculated as the mean of the incidence values recorded from 2000 to 2024. For age-specific incidence rate calculations, patients were categorized into the following age groups: 0–9, 10–19, 20–29, 30–39, 40–49, 50–59, 60–69, 70–79, and ≥ 80. Sex-specific incidence rates were calculated separately for males and females. Between-subgroup differences were assessed using a one-way analysis of variance (ANOVA). The effect sizes of these differences were summarized as eta-squared (η) and interpreted using conventional benchmarks: < 0.01 as negligible, < 0.06 as small, < 0.14 as medium, and ≥ 0.14 as large. The two-sided p-values of the post-hoc pairwise comparisons of mean difference (MD) among various age subgroups were adjusted using Bonferroni correction.

## Results

### Characteristics of study cohort

Our study samples comprised 36,970 patients presenting with tendinopathy (n = 27,838) and/or non-traumatic tendon rupture (n = 9,946). Elbow tendinopathies emerged as the most common condition, affecting 57.3% of all participants with tendinopathy (n = 15,946). Foot and ankle tendinopathies were observed in 22.8% (n = 6,334), while shoulder tendinopathies were diagnosed in 12.9% (n = 3,605). Hand and wrist tendinopathy was documented in 3.6% (n = 998), and knee tendinopathies were reported in 3.4% (n = 943). Hip tendinopathies were relatively rare, impacting < 0.1% (n = 12). In contrast, shoulder tendon ruptures occurred in 41.9% (n = 4,165) among all participants with non-traumatic tendon rupture, foot and ankle tendon ruptures were noted in 32.7% (n = 3,252), hand and wrist tendon ruptures were identified in 18.7% (n = 1,862), and knee tendon ruptures were reported in 6.7% (n = 667).

### Total incidences of tendon conditions

The overall annual incidence rate for the 17 included tendinopathies and non-traumatic tendon ruptures was 21.05 ± 7.08 cases per 100,000 persons. Specifically, the overall annual incidence rate for the 10 tendinopathies was 15.48 ± 6.33, while that for the 7 non-traumatic tendon ruptures was 5.57 ± 0.93.

### Incidences of tendinopathy

**Fig. 1:**
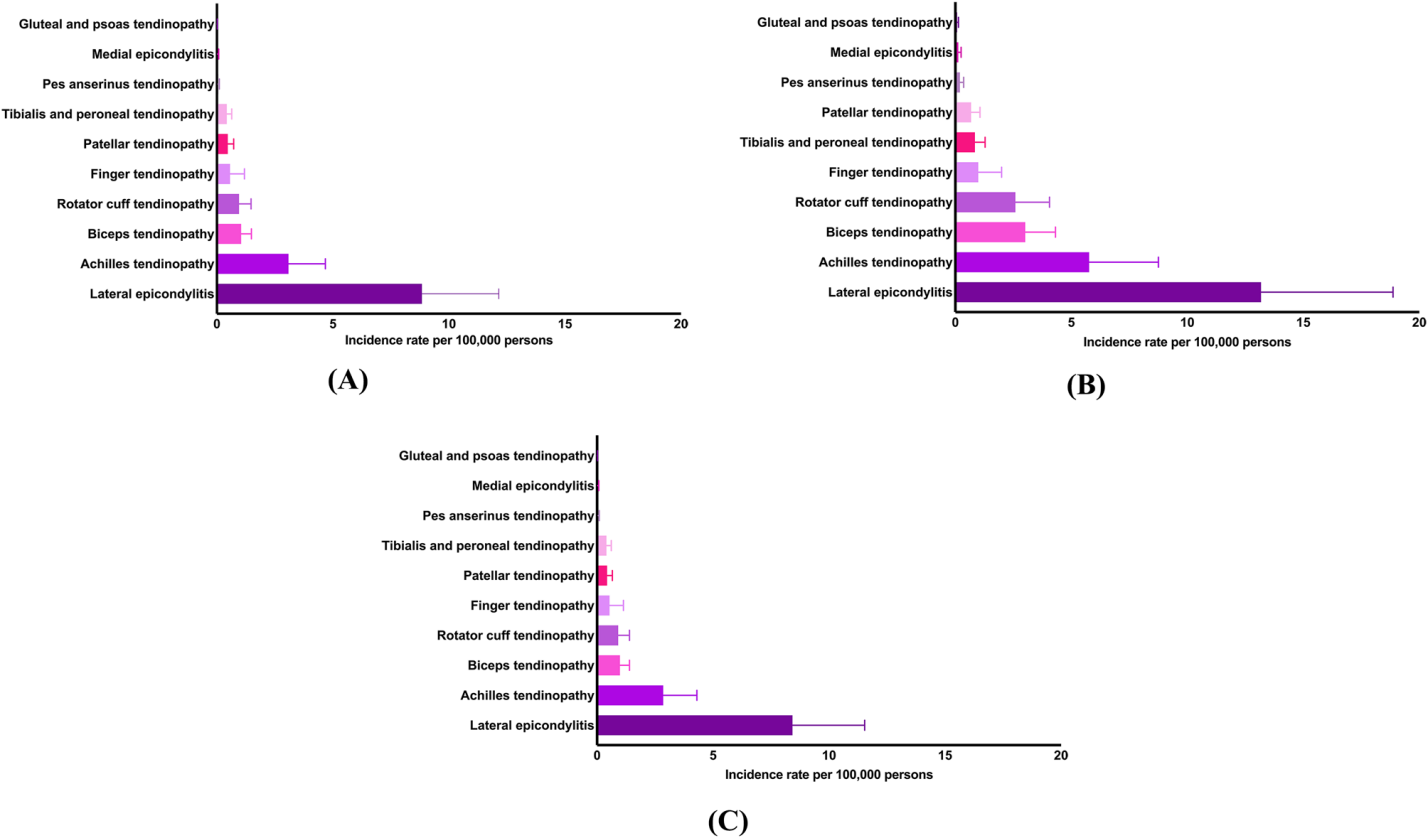
(**A**) Overall annual incidence rate of tendinopathies from the years 2000 to 2024. Mean ± standard deviation. (**B**) Age-standardized annual incidence rate of tendinopathies from the years 2000 to 2024. Mean ± standard deviation. (**C**) Sex-standardized annual incidence rate of tendinopathies from the years 2000 to 2024. Mean ± standard deviation.

*Overall rates*. Tendinopathy exhibited the highest overall annual incidence in cases of lateral epicondylitis (Fig. 1A), with a rate of 8.84 ± 3.31 cases per 100,000 persons. This was followed by Achilles tendinopathy (3.09 ± 1.59) and biceps tendinopathy (1.04 ± 0.44). Other conditions included rotator cuff tendinopathy (0.95 ± 0.52), finger tendinopathy (0.56 ± 0.63), patellar tendinopathy (0.47 ± 0.25), and tibialis and peroneal tendinopathy (0.43 ± 0.22). Less common forms comprised pes anserinus tendinopathy (0.06 ± 0.04), medial epicondylitis (0.03 ± 0.04), and gluteal and psoas tendinopathy (0.01 ± 0.01).

*Age-standardized rates*. Lateral epicondylitis remained the commonest tendinopathy, at 13.18 ± 5.69 cases per 100,000 persons, followed by Achilles tendinopathy at 5.77 ± 2.98 (Fig. 1B). Biceps tendinopathy was reported at 3.01 ± 1.30 and rotator cuff tendinopathy at 2.59 ± 1.47. Lesser frequencies were observed for finger tendinopathy (0.99 ± 1.01), tibialis and peroneal tendinopathy (0.84 ± 0.44), and patellar tendinopathy (0.68 ± 0.39). The lowest rates were seen in the pes anserinus (0.20 ± 0.16), medial epicondylitis (0.13 ± 0.12), and gluteal and psoas tendinopathy (0.08 ± 0.05).

*Sex-standardized rates*. Similar trends were reflected (Fig. 1C), with lateral epicondylitis having the topmost rate at 8.42 ± 3.12 cases per 100,000 persons, followed by Achilles tendinopathy (2.85 ± 1.45), biceps tendinopathy (0.99 ± 0.41), and rotator cuff tendinopathy (0.91 ± 0.49). Other less frequent conditions included finger (0.54 ± 0.60), patellar (0.43 ± 0.23), and tibialis and peroneal tendinopathy (0.40 ± 0.21), while pes anserinus (0.05 ± 0.04), medial epicondylitis (0.03 ± 0.04), and gluteal and psoas tendinopathy (0.01 ± 0.01) were relatively rare.

### Incidences of non-traumatic tendon rupture

**Fig. 2:**
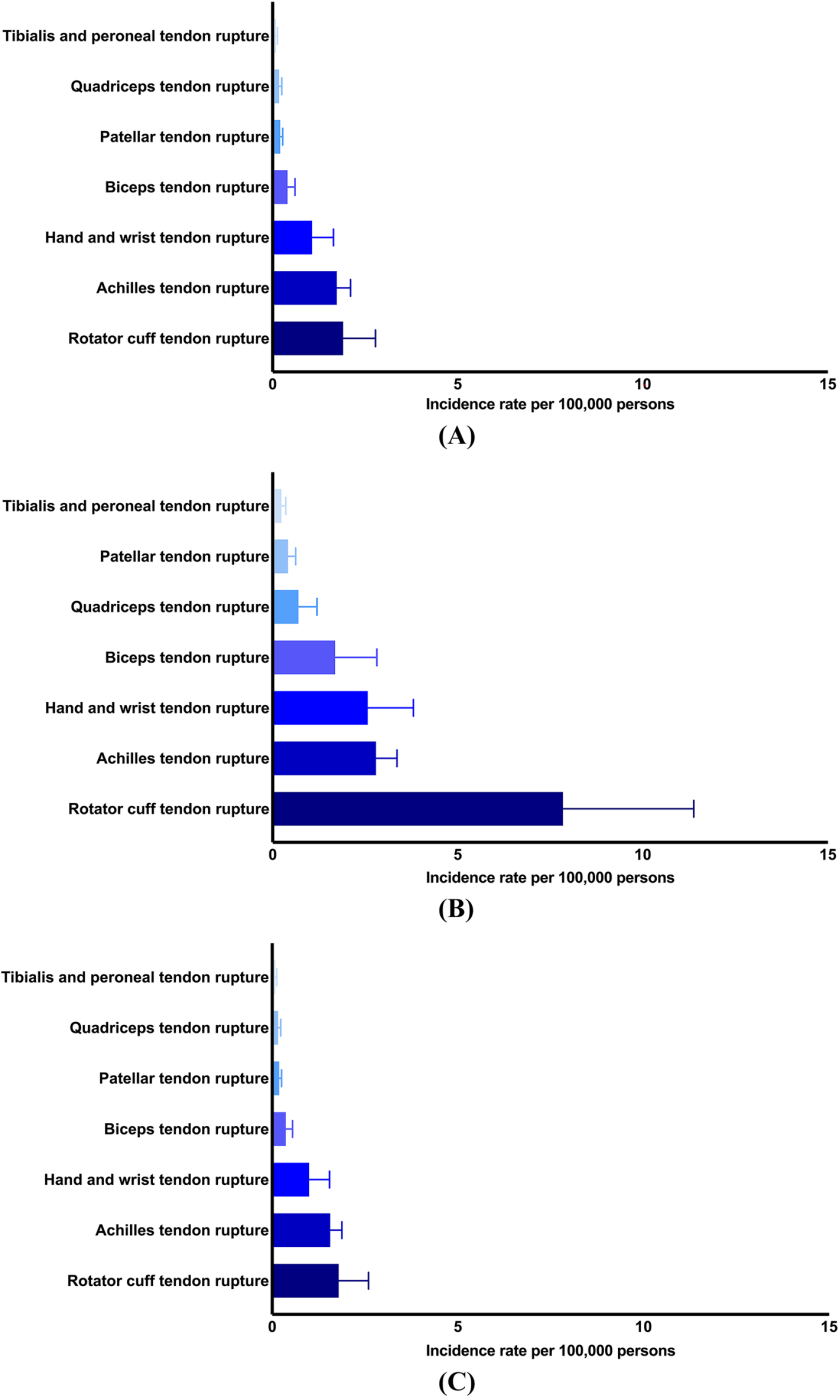
(**A**) Overall annual incidence rate of non-traumatic tendon ruptures from the years 2000 to 2024. Mean ± standard deviation. (**B**) Age-standardized annual incidence rate of non-traumatic tendon ruptures from the years 2000 to 2024. Mean ± standard deviation. (**C**) Sex-standardized annual incidence rate of non-traumatic tendon ruptures from the years 2000 to 2024. Mean ± standard deviation.

*Overall rates*. Rotator cuff tendon ruptures had the highest overall annual incidence at 1.90 ± 0.87 cases per 100,000 persons (Fig. 2A). Achilles tendon ruptures were followed closely at 1.73 ± 0.37, whereas hand and wrist tendon ruptures were charted at 1.07 ± 0.58. Additional rupture types reported included biceps (0.40 ± 0.20), patellar (0.21 ± 0.07), quadriceps (0.17 ± 0.08), and tibialis and peroneal tendon ruptures (0.09 ± 0.04).

*Age-standardized rates*. Rotator cuff tendon ruptures were the most common at 7.84 ± 3.53 cases per 100,000 persons (Fig. 2B), followed by Achilles tendon ruptures (2.79 ± 0.57) and hand and wrist tendon ruptures (2.57 ± 1.23). Reports for biceps tendon ruptures were at 1.69 ± 1.13, quadriceps at 0.69 ± 0.50, patellar at 0.41 ± 0.21, and tibialis and peroneal at 0.23 ± 0.12.

*Sex-standardized rates*. The highest incidence was observed for rotator cuff tendon ruptures at 1.79 ± 0.80 cases per 100,000 persons (Fig. 2C), followed by Achilles tendon ruptures (1.56 ± 0.31) and hand and wrist tendon ruptures (1.00 ± 0.55). Biceps tendon ruptures were recorded at 0.37 ± 0.18, while the incidences of patellar (0.19 ± 0.06), quadriceps (0.16 ± 0.07), and tibialis and peroneal tendon ruptures (0.08 ± 0.04) were comparatively lower.

### Age effects on incidence of tendinopathy

**Fig. 3:**
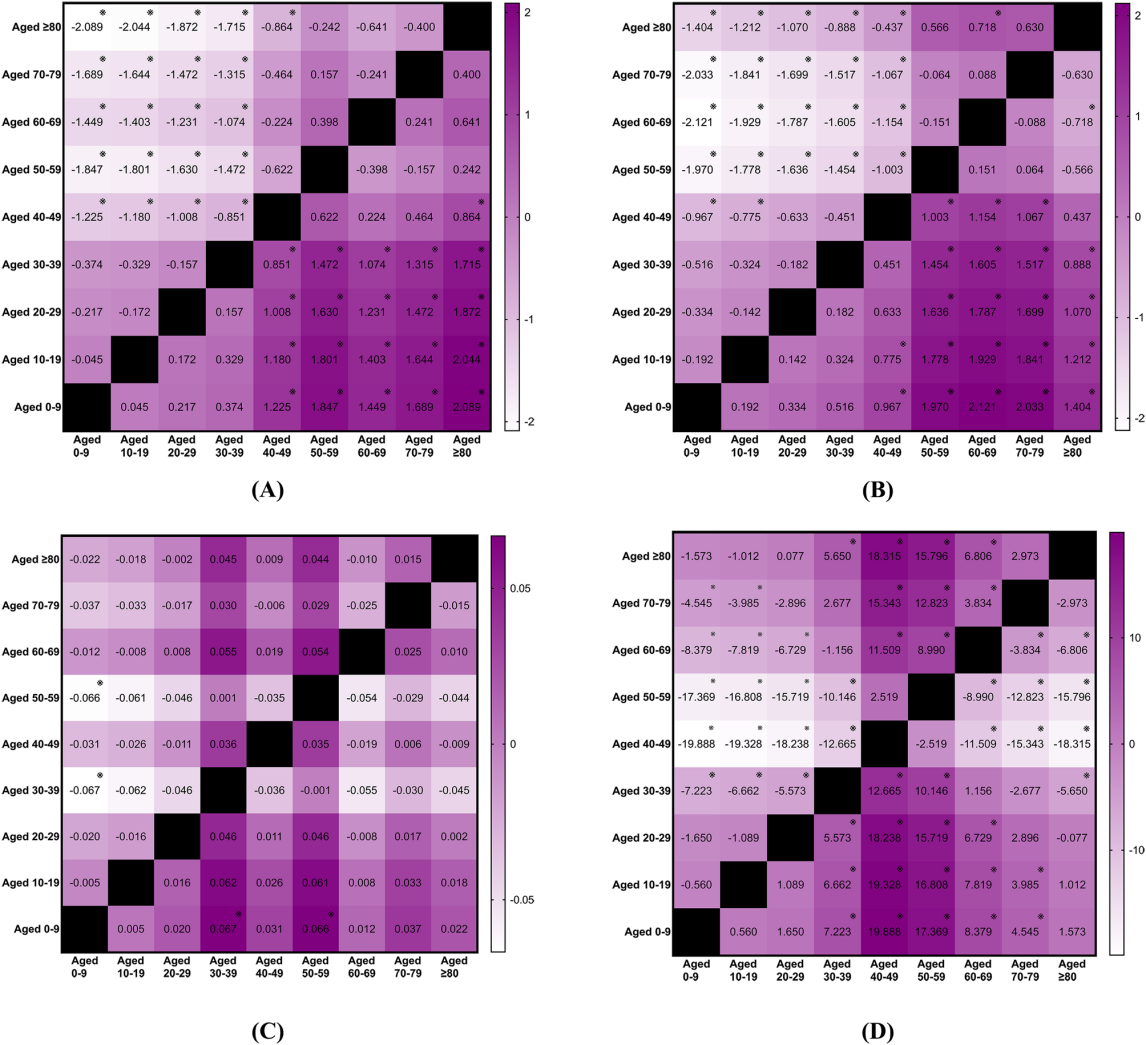

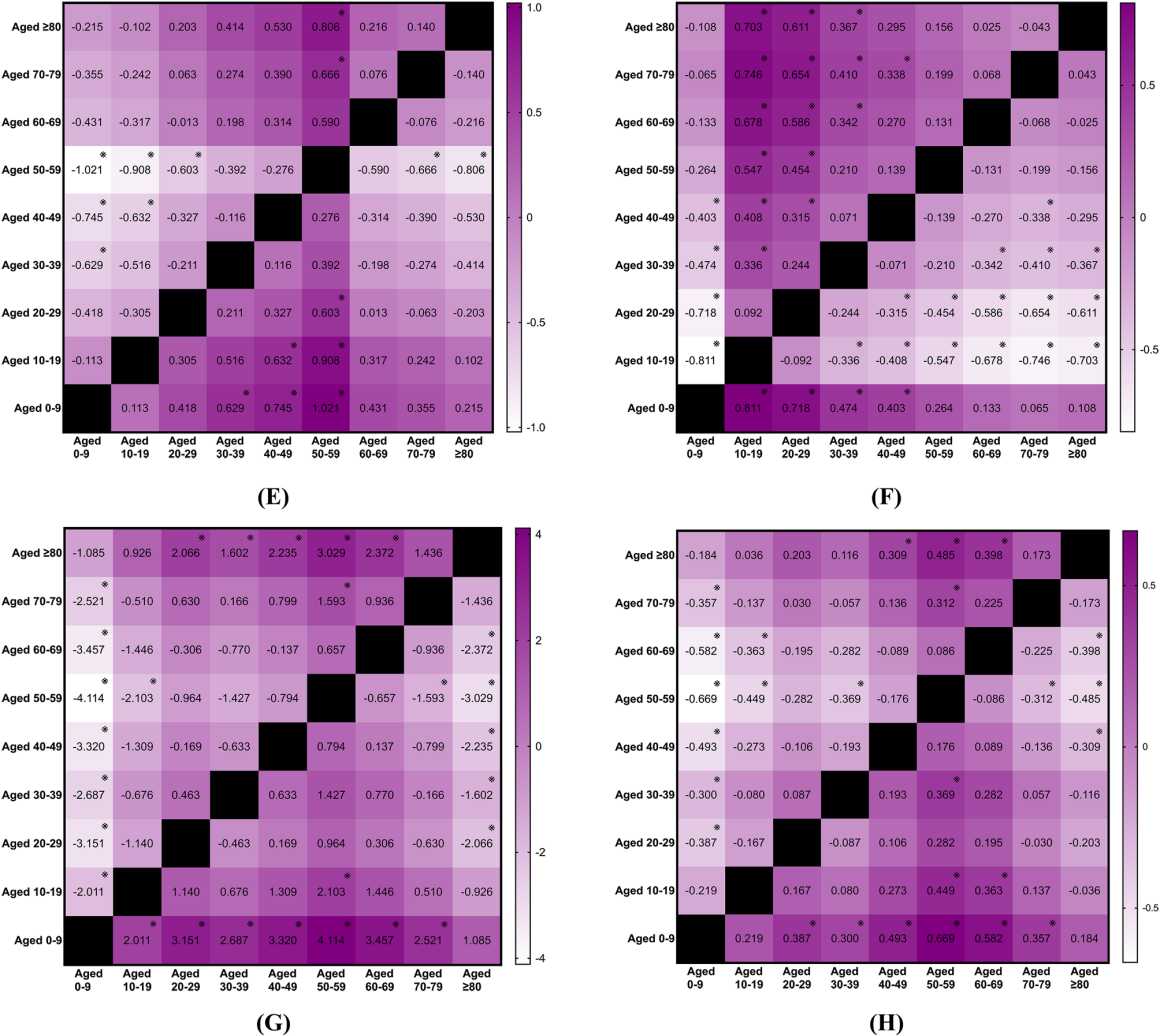
(**A**) Mean difference in age-specific annual incidence rates of rotator cuff tendinopathy between the years 2000 and 2024, categorized by age groups. Mean difference is calculated by subtracting the group in column from the group in row, ※ as significant. (**B**) Mean difference in age-specific annual incidence rates of biceps tendinopathy between the years 2000 and 2024, categorized by age groups. Mean difference is calculated by subtracting the group in column from the group in row, ※ as significant. (**C**) Mean difference in age-specific annual incidence rates of medial epicondylitis between the years 2000 and 2024, categorized by age groups. Mean difference is calculated by subtracting the group in column from the group in row, ※ as significant. (**D**) Mean difference in age-specific annual incidence rates of lateral epicondylitis between the years 2000 and 2024, categorized by age groups. Mean difference is calculated by subtracting the group in column from the group in row, ※ as significant. (**E**) Mean difference in age-specific annual incidence rates of finger tendinopathy between the years 2000 and 2024, categorized by age groups. Mean difference is calculated by subtracting the group in column from the group in row, ※ as significant. (**F**) Mean difference in age-specific annual incidence rates of patellar tendinopathy between the years 2000 and 2024, categorized by age groups. Mean difference is calculated by subtracting the group in column from the group in row, ※ as significant. (**G**) Mean difference in age-specific annual incidence rates of Achilles tendinopathy between the years 2000 and 2024, categorized by age groups. Mean difference is calculated by subtracting the group in column from the group in row, ※ as significant. (**H**) Mean difference in age-specific annual incidence rates of tibialis and peroneal tendinopathy between the years 2000 and 2024, categorized by age groups. Mean difference is calculated by subtracting the group in column from the group in row, ※ as significant.

Statistically significant age effects were observed regarding the age-specific annual incidence rates for rotator cuff tendinopathy (p < 0.001; η^2^ = 0.546, large effect), biceps tendinopathy (p < 0.001; η^2^ = 0.575, large effect), medial epicondylitis (p = 0.005; η^2^ = 0.096, medium effect), lateral epicondylitis (p < 0.001; η^2^ = 0.763, large effect), finger tendinopathy (p < 0.001; η^2^ = 0.181, large effect), patellar tendinopathy (p < 0.001; η^2^ = 0.408, large effect), Achilles tendinopathy (p < 0.001; η^2^ = 0.354, large effect), and tibialis and peroneal tendinopathy (p < 0.001; η^2^ = 0.292, large effect). The incidence rates for rotator cuff and biceps tendinopathy increased with advancing age, peaking in older age groups, specifically those aged 40 and above (Figs. 3A-B). Among patients suffering from medial epicondylitis, those aged 30–39 and 50–59 presented higher incidence rates than their counterparts (Fig. 3C). In the case of lateral epicondylitis, incidence rates also increased with age, reaching a peak in the middle-aged group, specifically those aged 40–59 (Fig. 3D). The highest incidence of finger tendinopathy was observed in individuals aged 50–59 (Fig. 3E). For patellar tendinopathy, the highest incidence was noted in the age group of 10–29 (Fig. 3F). The incidence of Achilles tendinopathy was highest among individuals aged 50–59 (Fig. 3G), while tibialis and peroneal tendinopathy also showed the highest incidence in this age group (Fig. 3H). There was no significant mean difference in incidence across age groups for patients with gluteal and psoas tendinopathy (p = 0.496) or pes anserinus tendinopathy (p = 0.122).

### Age effects on incidence of non-traumatic tendon rupture

**Fig. 4:**
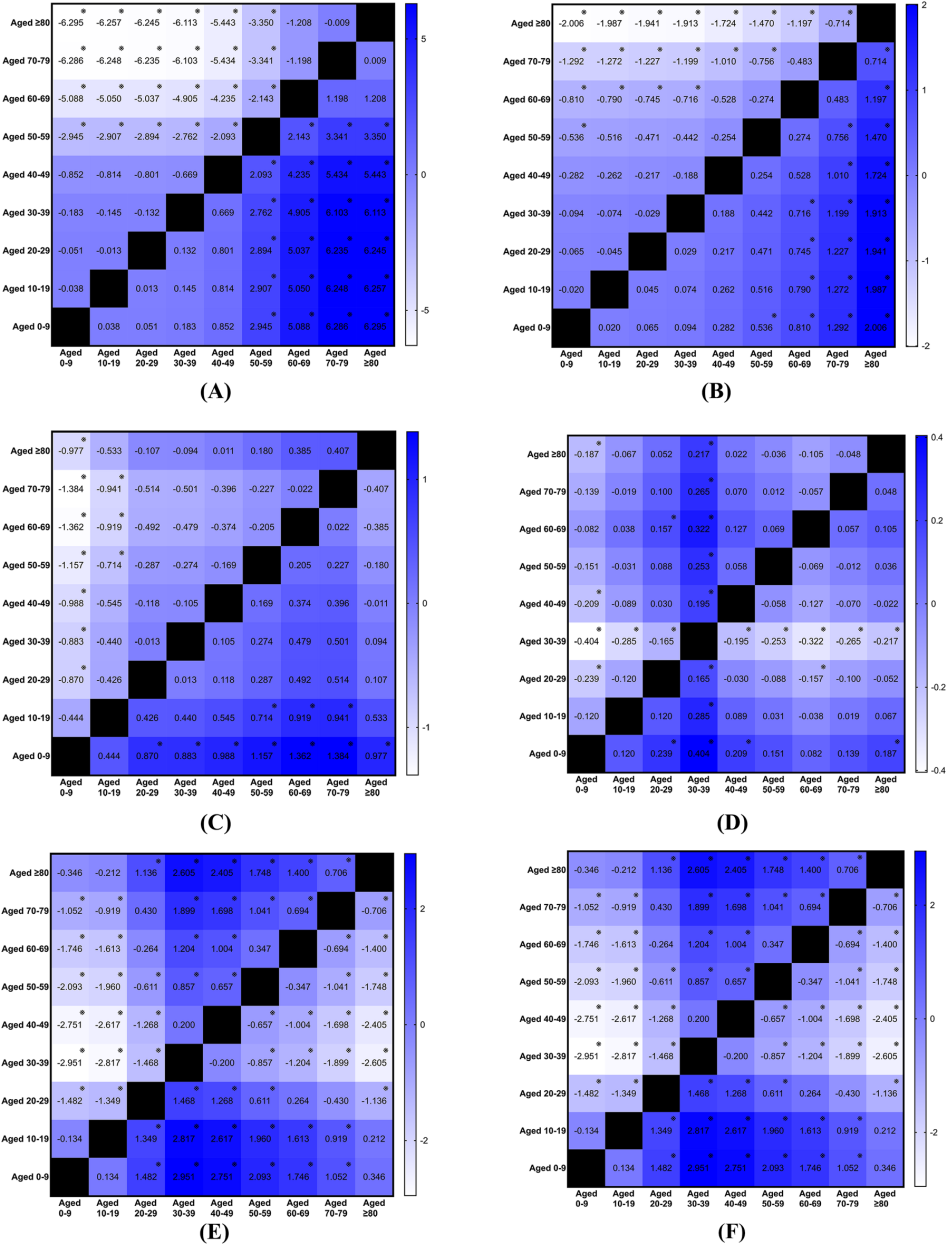
(**A**) Mean difference in age-specific annual incidence rates of rotator cuff tendon rupture between the years 2000 and 2024, categorized by age groups. Mean difference is calculated by subtracting the group in column from the group in row, ※ as significant. (**B**) Mean difference in age-specific annual incidence rates of biceps tendon rupture between the years 2000 and 2024, categorized by age groups. Mean difference is calculated by subtracting the group in column from the group in row, ※ as significant. (**C**) Mean difference in age-specific annual incidence rates of hand and wrist tendon rupture between the years 2000 and 2024, categorized by age groups. Mean difference is calculated by subtracting the group in column from the group in row, ※ as significant. (**D**) Mean difference in age-specific annual incidence rates of patellar tendon rupture between the years 2000 and 2024, categorized by age groups. Mean difference is calculated by subtracting the group in column from the group in row, ※ as significant. (**E**) Mean difference in age-specific annual incidence rates of quadriceps tendon rupture between the years 2000 and 2024, categorized by age groups. Mean difference is calculated by subtracting the group in column from the group in row, ※ as significant. (**F**) Mean difference in age-specific annual incidence rates of Achilles tendon rupture between the years 2000 and 2024, categorized by age groups. Mean difference is calculated by subtracting the group in column from the group in row, ※ as significant.

Similarly, there were statistical significant effects related to age detected concerning the age-specific annual incidence rates for rotator cuff tendon rupture (p < 0.001; η^2^ = 0.754, large effect), biceps tendon rupture (p < 0.001; η^2^ = 0.572, large effect), hand and wrist tendon rupture (p < 0.001; η^2^ = 0.260, large effect), patellar tendon rupture (p < 0.001; η^2^ = 0.294, large effect), quadriceps tendon rupture (p < 0.001; η^2^ = 0.280, large effect), and Achilles tendon rupture (p < 0.001; η^2^ = 0.808, large effect). For individuals with rotator cuff tendon rupture, those aged 50 and above exhibited higher incidence rates than their younger counterparts (Fig. 4A). In cases of biceps tendon rupture, those aged 60 and above demonstrated higher incidence rates than younger age groups (Fig. 4B). The incidence of hand and wrist tendon rupture was highest among individuals aged 60–79 (Fig. 4C). For patellar tendon rupture, the age group of 30–39 had the highest incidence (Fig. 4D). The highest incidence of quadriceps tendon rupture was noted in the 60–79 age range (Fig. 4E). Lastly, individuals aged 30–49 exhibited the highest incidence of Achilles tendon rupture (Fig. 4F). No significant difference in incidence was observed across age groups for patients with tibialis and peroneal tendon rupture (p = 0.077).

### Sex effects on incidence of tendinopathy

**Fig. 5:**
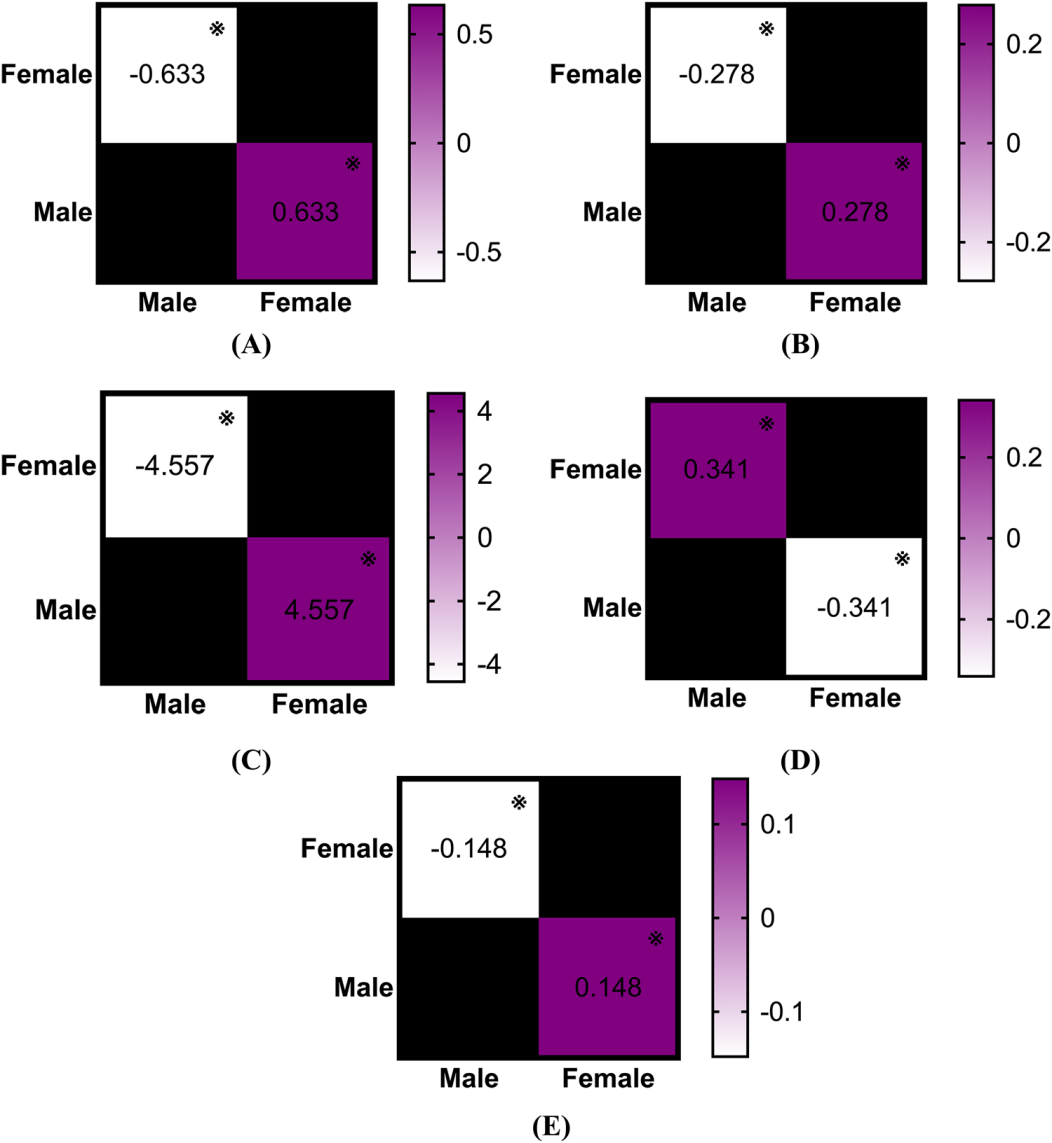
(**A**) Comparison of sex-specific annual incidence rates of rotator cuff tendinopathy between the years 2000 and 2024. Mean difference is calculated by subtracting the group in column from the group in row, ※ as significant. (**B**) Comparison of sex-specific annual incidence rates of biceps tendinopathy between the years 2000 and 2024. Mean difference is calculated by subtracting the group in column from the group in row, ※ as significant. (**C**) Comparison of sex-specific annual incidence rates of lateral epicondylitis between the years 2000 and 2024. Mean difference is calculated by subtracting the group in column from the group in row, ※ as significant. (**D**) Comparison of sex-specific annual incidence rates of patellar tendinopathy between the years 2000 and 2024. Mean difference is calculated by subtracting the group in column from the group in row, ※ as significant. (**E**) Comparison of sex-specific annual incidence rates of tibialis and peroneal tendinopathy between the years 2000 and 2024. Mean difference is calculated by subtracting the group in column from the group in row, ※ as significant.

The incidence rate of various types of tendinopathy was compared between males and females. Females exhibited a significantly higher incidence of rotator cuff tendinopathy (p < 0.001; η^2^ = 0.294, large effect; Fig. 5A), biceps tendinopathy (p = 0.026; η^2^ = 0.099, medium effect; Fig. 5B), lateral epicondylitis (p < 0.001; η^2^ = 0.347, large effect; Fig. 5C), and tibialis and peroneal tendinopathy (p = 0.022; η^2^ = 0.105, medium effect; Fig. 5E) compared to males. Conversely, males demonstrated a significantly higher incidence of patellar tendinopathy than females (p < 0.001; η^2^ = 0.294, large effect; Fig. 5D). For conditions including medial epicondylitis (p = 0.785), finger tendinopathy (p = 0.171), gluteal and psoas tendinopathy (p = 0.212), pes anserinus tendinopathy (p = 0.550), and Achilles tendinopathy (p = 0.070), no significant differences were observed between sexes.

### Sex effects on incidence of non-traumatic tendon rupture

**Fig. 6:**
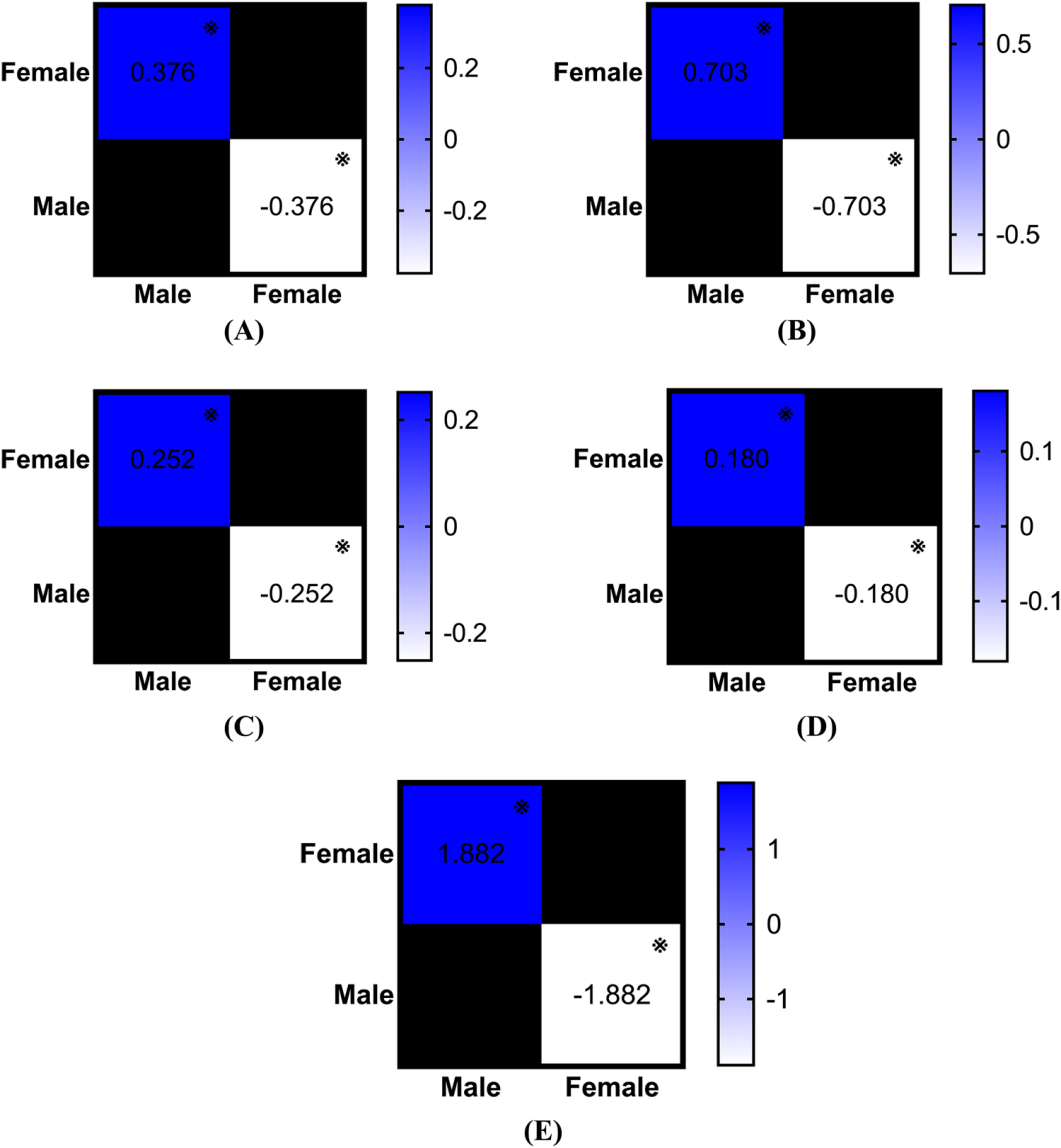
(**A**) Comparison of sex-specific annual incidence rates of biceps tendon rupture between the years 2000 and 2024. Mean difference is calculated by subtracting the group in column from the group in row, ※ as significant. (**B**) Comparison of sex-specific annual incidence rates of hand and wrist tendon rupture between the years 2000 and 2024. Mean difference is calculated by subtracting the group in column from the group in row, ※ as significant. (**C**) Comparison of sex-specific annual incidence rates of patellar tendon rupture between the years 2000 and 2024. Mean difference is calculated by subtracting the group in column from the group in row, ※ as significant. (**D**) Comparison of sex-specific annual incidence rates of quadriceps tendon rupture between the years 2000 and 2024. Mean difference is calculated by subtracting the group in column from the group in row, ※ as significant. (**E**) Comparison of sex-specific annual incidence rates of Achilles tendon rupture between the years 2000 and 2024. Mean difference is calculated by subtracting the group in column from the group in row, ※ as significant.

Interestingly, the situation regarding non-traumatic tendon rupture is entirely different. Males exhibited a significantly higher incidence of biceps tendon rupture (p < 0.001; η^2^ = 0.451, large effect; Fig. 6A), hand and wrist tendon rupture (p < 0.001; η^2^ = 0.241, large effect; Fig. 6B), patellar tendon rupture (p < 0.001; η^2^ = 0.627, large effect; Fig. 6C), quadriceps tendon rupture (p < 0.001; η^2^ = 0.489, large effect; Fig. 6D), and Achilles tendon rupture (p < 0.001; η^2^ = 0.815, large effect; Fig. 6E) compared to females. For rotator cuff (p = 0.131) and tibialis and peroneal tendon rupture (p = 0.144), no differences were found between sexes.

### Relationship between tendinopathy and non-traumatic tendon rupture

The time interval between these events was calculated for patients with a prior diagnosis of tendinopathy who subsequently experienced tendon rupture. Only 0.3% of patients with non-traumatic rotator cuff tendon rupture had a history of rotator cuff tendinopathy (n = 11), with a mean duration of 22 ± 33 months between diagnoses. Similarly, 0.3% of patients with non-traumatic biceps tendon rupture had a prior biceps tendinopathy (n = 2), with an average interval of 24 ± 32 months. In the case of patellar tendon rupture, 0.3% of patients had a history of patellar tendinopathy (n = 1), with an average interval of 75 ± 0 months between diagnoses. Additionally, 0.8% of patients with patellar tendon rupture had a history of patellar tendinopathy (n = 26), with a mean interval of 40 ± 41 months. Finally, 2.0% of patients with patellar tendon rupture had prior patellar tendinopathy (n = 3), with an average interval of 37 ± 32 months between diagnoses.

## Discussion

### Summary of findings

The present study addressed the epidemiology of 10 tendinopathies and 7 non-traumatic tendon ruptures, encompassing a cohort of 36,970 patients from 47 local public hospitals over 25 years. The annual incidence of tendinopathy was highest in the elbow, while non-traumatic tendon ruptures were predominantly observed in the shoulder. Middle age (40–59) was associated with a heightened incidence of most tendinopathies, except patellar and Achilles tendinopathy. Female patients displayed higher incidences of several tendinopathies, whereas male patients exhibited greater incidences of patellar tendinopathy along with various non-traumatic tendon ruptures.

### Comparisons with relevant contemporary data on tendinopathy

Our analysis of various tendinopathies revealed notable discrepancies in incidence rates. In contrast to the existing literature, the incidence rates for tendinopathy showed substantial variability across studies, likely because most used a cross-sectional design.

*Achilles tendinopathy*. While Kujala et al. reported an incidence rate of 4,808 per 100,000 individuals from the Finnish general population (n = 416)^18^, this sample size is deemed inadequate. Jonge et al. identified a rate of 185 per 100,000 in the Netherlands, defined solely by all patients who visited the clinics (n = 57,725)^19^. Albers et al. documented a similar rate of 216 per 100,000, again limited to a Dutch clinic population (n = 10,651)^21^. Riel et al. recorded an incidence of 170 per 100,000 in Denmark, also based on clinic attendees (n = 147)^22^. However, it is essential to note that these three studies relied exclusively on hospital visit populations, which may have led to an overestimation of the true incidence rate. Conversely, Lagas et al. followed a group of runners over a short period and reported an elevated rate of 5,184 per 100,000 in the Netherlands (n = 1,929)^20^. Bittencourt et al. noted an annual rate of 580 per 100,000 in a Brazilian sports club (n = 1,553)^23^. Both studies focused on athlete populations, which are known to be at high risk, thus their findings could not be generalized to the broader population.

*Patellar tendinopathy*. Albers et al. reported a rate of 160 per 100,000^21^, while Riel et al. found a rate of 50 per 100,000^22^, and Bittencourt et al. documented a higher rate of 5,087 per 100,000^23^.

*Hip tendinopathy*. Albers et al. reported 113 per 100,000^21^, Riel et al. reported no cases^22^, and Bittencourt et al. reported a rate of 773 per 100,000^23^.

*Shoulder tendinopathy*. Only one study, Kettunen et al. reported a rate of 4,567 per 100,000 individuals in the Finnish general population (n = 416)^34^, which also suffers from small samples.

*Explanation of variations between findings and literature*. Since this study employed large-scale samples, it enhanced the representativeness of the general population compared with smaller studies reported in the literature. Furthermore, the population at risk we used as the denominator encompasses the entire regional population, unlike some studies that only considered hospital attendees. Although this approach may yield lower incidence rates (only 75% of local hospitals were included), it is essential to note that our measures do not overestimate incidence. Rather, they provide an accurate estimate of the true situation, extending beyond the hospital setting.

### Comparisons with relevant contemporary data on tendon ruptures

Similarly, our data on non-traumatic ruptured tendons have revealed significant variations in incidence rates. Upon reviewing the existing literature, no studies have specifically examined non-traumatic tendon ruptures. All studies on tendon ruptures did not specify whether the ruptures were traumatic or non-traumatic, nor whether they were acute or chronic^24–33^.

### Possible mechanisms behind age effects

The current findings indicated that tendinopathies and non-traumatic tendon ruptures were more prevalent in specific age groups, demonstrating a general trend of increased incidence with advancing age for most conditions. This observation suggests that age represents a significant risk factor for tendinopathy and non-traumatic tendon rupture, likely attributable to cumulative wear and tear, reduced tendon elasticity, and various age-associated alterations in tendon structure and function^38^. In the context of rotator cuff and biceps tendinopathy, the elevated incidence in older age cohorts may result from repetitive overhead activities and degenerative changes within the tendons^3^. Similarly, medial and lateral epicondylitis exhibited heightened incidence rates among middle-aged individuals, likely attributable to repetitive strain and overuse^39^. Conversely, patellar tendinopathy was more prevalent in younger age groups, possibly correlating with athletic activities and high physical demands^40^. Likewise, Achilles tendinopathy primarily affected younger age groups, where participation in sports-related activities contributed to its development^41^.

### Potential mechanisms underlying sex differences

Moreover, the influence of sex on tendinopathy and non-traumatic tendon rupture has unveiled distinct patterns in incidence rates. This may be attributable to a combination of hormonal and biomechanical factors. While estrogen can promote tendon collagen synthesis that can be advantageous for tendon health and recovery in women, elevated levels of estrogen in active young female athletes may amplify injury risk due to diminished fibrillar crosslinking and increased joint laxity^42^. Furthermore, the mechanical strength of isolated tendon collagen fascicles is greater in men than in women, which may contribute to the higher incidence of tendon injuries in women during physical activity^43^.

### Clinical significance

Our investigation into tendinopathy and non-traumatic tendon rupture provides significant implications for population health and clinical practice. Notably, lateral epicondylitis, rotator cuff tears, and Achilles tendinopathy/rupture should be prioritized to mitigate associated financial and societal burdens. Moreover, prevention strategies should specifically target middle-aged adults (40–59 years) and females for tendinopathies, while males should be the focus for non-traumatic tendon ruptures due to their increased risk. Primary care physicians need to be equipped to recognize and address tendinopathies such as lateral epicondylitis and Achilles tendinopathy, facilitating timely referral to specialist services and preventing disease progression. Early intervention by orthopedic surgeons could further reduce the prevalence and strain on specialist and hospital resources. Conversely, non-traumatic tendon ruptures are predominantly managed in acute settings. Enhancing training for emergency department staff regarding the management of rotator cuff and Achilles tendon ruptures could improve care quality and efficiency. Nevertheless, it is important to acknowledge that our findings are context-specific, and their applicability to other populations requires further validation. Overall, this study emphasizes the urgent need for integrated preventative and clinical strategies to address these tendon conditions.

### Future direction

We lacked information on histological findings and imaging results, as well as on physiotherapy use and outcomes, and on clinical presentations such as pain levels and range of motion. This absence of detailed information limits our ability to establish robust biological and clinical linkages. Future studies should examine the associations between these factors and the site-, age-, and sex-specific incidences and prevalences of tendinopathy and tendon rupture to enhance the applicability of the findings.

## Limitations

A limitation of our study is the exclusion of a subset of local private hospitals (25%), which may affect the external validity of the population-based data collection. This exclusion could introduce selection bias, particularly if the omitted hospitals serve distinct patient populations or follow different clinical practices. Moreover, the low incidence of tendon ruptures with prior tendinopathy may be due to underreporting. In the meantime, the use of ICD-9-CM was conceptually limited by its lack of specificity and a small number of codes. These weaknesses made it inadequate for detailed clinical documentation, data analysis, and modern healthcare needs, prompting the shift to ICD-11. Additionally, the etiology of tendinopathy remains ambiguous, thereby complicating our comprehension of its development and progression. Similarly, the factors contributing to non-traumatic tendon rupture are not thoroughly understood, introducing an additional layer of complexity to our research. Nevertheless, our study contributes valuable insights into the epidemiology of tendon conditions, despite inherent challenges in fully capturing all associated factors. While age and sex were identified as risk factors, we acknowledge that other important modifiable factors (e.g., fluoroquinolone exposure, co-prescription with corticosteroids, obesity, diabetes, and rheumatoid arthritis) were not analyzed due to the unavailability of this information in the CDARS database. Future studies utilizing more comprehensive clinical databases with identifiable patient information, obtained with patient consent, would facilitate a more thorough investigation of these factors. Taken together, these limitations suggest that while our findings offer valuable insights, they should be interpreted with caution.

## Conclusions

This population-based study examined the epidemiology of 10 different tendinopathies and 7 types of non-traumatic tendon ruptures, involving a cohort of 36,970 patients from 47 local public hospitals over a 25-year period. Tendinopathy most frequently occurred in the elbow, while non-traumatic tendon ruptures mainly happened in the shoulder. Most tendinopathies increased with age, but patellar and Achilles issues were more common in younger people. Females had higher rates of several tendinopathies. Males only showed higher rates of patellar tendinopathy, along with most non-traumatic ruptures. Targeted interventions should be implemented as a public health strategy for high-burden tendon conditions. Primary care providers could enhance early referral processes, orthopedists may consider earlier interventions, and emergency staff may benefit from additional training to improve management efficiency.

## Data Availability

The data supporting the study findings can be obtained from the corresponding author upon reasonable request.
